# Plant detritus is selectively consumed by estuarine copepods and can augment their survival

**DOI:** 10.1038/s41598-019-45503-6

**Published:** 2019-06-24

**Authors:** Jennifer Harfmann, Tomofumi Kurobe, Brian Bergamaschi, Swee Teh, Peter Hernes

**Affiliations:** 1 Department of Land, Air, and Water Resources, University of California, Davis, CA USA; 2 Department of Anatomy, Physiology, and Cell Biology, School of Veterinary Medicine, University of California, Davis, CA USA; 3 United States Geological Survey, Sacramento, CA USA

**Keywords:** High-throughput screening, Carbon cycle, Restoration ecology

## Abstract

Particulate material comprising the detrital remains of terrestrial plants and macrophytes is a substantial source of organic matter to estuaries and therefore has the potential to support the energy demands of the pelagic aquatic food web. Despite the prevalence of macrophytic or terrestrial particulate organic carbon (tPOC), phytoplankton are nutritionally superior and are thought to be the primary food resource for zooplankton. However, estuarine phytoplankton primary productivity abundances can wax and wane, and often production cannot meet heterotrophic energy needs. In this study, we examined how tPOC (detritus of macrophytes and grasses) may affect survival of a calanoid copepod (*Eurytemora affinis*) common in the San Francisco Estuary (SFE), an estuary with relatively low phytoplankton primary productivity. Using chemical biomarkers and a targeted DNA metagenomic methodology, we show that *E. affinis* consumed tPOC (dominated by *Schoenoplectus* sp., or tule) even when phytoplankton were abundant and tPOC was scarce. Furthermore, we found that a mixed diet of phytoplankton and terrestrial material (1:3 carbon ratio) enhanced the survival of *E. affinis* over a diet of phytoplankton alone. These data show that tPOC can be a vital supplementary food source for zooplankton, perhaps extending survival during low phytoplankton periods, and may help explain elevated zooplankton abundances in tidal wetlands and other detrital-dominated regions.

## Introduction

In most estuarine systems worldwide, phytoplankton primary production is perceived as the major food source for zooplankton, effectively fueling higher trophic level energy needs^1–3^. Terrestrial particulate organic carbon (tPOC, herein including macrophytes) loading is often overlooked as a subsidy for zooplankton diet, even in cases where phytoplankton growth is limited^4,5^. However, even in systems with abundant autochthonous primary production, spatial and temporal heterogeneity create zones and time periods in which algal resources are low and tPOC dominates the bioavailable organic matter (OM) pool^6^. Therefore, the relevance of tPOC to food webs may be understated in many aquatic systems, but particularly in algal-limited estuaries.

The San Francisco Estuary (SFE) is just one example; high levels of turbidity decrease light penetration and limit algal growth, so much so that phytoplankton primary production in the SFE is ranked in the lowest 15% of the world’s estuaries^7^. River-borne allochthonous sources contribute five times more carbon than phytoplankton in this system and constitute the majority of OM in estuaries worldwide^8^. Since the contribution of terrestrial material and macrophyte-derived particulates often swamps autochthonous primary production in estuaries, we sought to examine the potential impact of grasses and macrophyte detrital material on pelagic aquatic food webs.

Despite its prevalence in the OM pool, tPOC itself is chemically inferior as a food resource for zooplankton. Vascular plants contain 5 to 25 times less protein than phytoplankton and lack essential polyunsaturated fatty acids (PUFAs) that zooplankton cannot produce themselves^9,10^. Plant material also contains much higher concentrations of aromatic macromolecules such as lignin and tannin that were traditionally regarded as inaccessible to the food web. We now know that these polymers are not inherently unable to be degraded biotically and instead their biolability is dependent on environmental factors, one of which is the presence or absence of other labile compounds, a phenomenon known as co-metabolism^11,12^. This may explain why cladocerans such as *Daphnia magna* are able to incorporate tPOC into their diets much more easily when labile phytoplankton are also present^13–15^.

In the SFE and other estuaries, copepods – not cladocerans – are the main link between primary producers and fish^16,17^ so investigating copepod diet is the key to unlocking the role of tPOC in the estuarine food web as a whole. Copepods are highly selective feeders, possessing the ability to perceive, capture, and ingest or reject their prey based on size and, in some cases, quality (i.e. avoidance of toxic food)^18–20^. Co-metabolism may therefore be part of copepod routine feeding behavior when labile phytoplankton are scarce, providing a major pathway through which macrophytic and terrestrial material becomes bioavailable.

The toolkit for detecting zooplankton consumption of tPOC is diverse and well-developed but also outdated. It includes a host of chemical biomarkers such as gut carbohydrate, lipid, and protein composition^14^ or stable isotopes^21^ to track plant material. Lignin is another chemical tool for this purpose, as it is unique to vascular plants and can provide source information (e.g. gymnosperm or angiosperm, woody or non-woody tissues). While such techniques have been suitable for detecting tPOC to-date, recent advancements in the field of metagenomics provide an opportunity to perform diet analysis with higher specificity and sensitivity. Originally developed to explore the microbiome in humans^22^, DNA metagenomics has recently taken hold as a method to explore zooplankton diet preferences, but such analyses have largely been focused on phytoplankton taxa^23,24^. Adding DNA metagenomic techniques to the tPOC toolkit can provide higher sensitivity zooplankton diet analyses than can be obtained by chemical analyses alone.

Here, we present results from feeding experiments with the calanoid copepod *Eurytemora affinis* with the hypotheses that (1) copepods selectively consume tPOC, and (2) tPOC is valuable to copepods and extends their survival in the presence of phytoplankton, identified herein as non-vascular autotrophs. Although we supplemented with more classic methods, we primarily used amplicon metagenomic analysis (hereinafter: metagenomic analysis) to unambiguously demonstrate copepod ingestion of tPOC, and we designed diet-controlled feeding experiments under phytoplankton-limited conditions to assess copepod survival.

## Methods

### Copepod cultures

Cultured *E. affinis* from the SFE, CA, USA have been maintained in the Aquatic Health Program Laboratory (AHP) at the University of California, Davis (UC Davis) since 2006. Copepods were raised in 120-L conical tanks in moderately hard reconstituted water^25^ adjusted to a salinity of 2 ppt using Instant Ocean Sea Salt (Pentair Aquatic Ecosystems, Inc.). Cultures were maintained at a temperature of 20 ± 2 °C with a 16:8 hour light:dark cycle and continuous aeration. Prior to feeding experiments, copepods were fed daily with 475 µg C L^−1^ day^−1^ of instant algae – equal volumes of Nannochloropsis 3600 (Eustigmatophyceae) and Pavlova (Prymnesiophyceae) from Reed Mariculture – based on protocols developed by the UC Davis AHP. Twice weekly, approximately one-third of the culture water was removed and replenished with clean reconstituted water to maintain water quality.

### Consumption feeding experiment with estuary water

To detect copepod consumption of autotrophs (both tPOC and phytoplankton), a cohort of cultured *E. affinis* was incubated with field water for four days, and both water quality and gut content were analyzed before and after feeding using chemical biomarkers (chlorophyll *a* and lignin as proxies for phytoplankton and tPOC, respectively) and DNA metagenomics.

Estuarine field water was collected from Suisun Marsh, CA, the largest brackish tidal marsh west of the Mississippi River. Suisun Marsh is comprised mainly of high stand *Schoenoplectus* sp. (tule) and impounded duck clubs, and contains a salinity control gate that operates seasonally to manage salinity in the region. Surface grab samples (pH = 7.6, salinity = 4.5‰) were collected in northeast Suisun Marsh (38.188°N, −121.976°W) in September 2017, when the salinity control gate was open. Field water was passed through a 63 µm sieve to filter out larger particulates that are indigestible to calanoid copepods^26^ and was transported to the lab in the dark on ice. A cohort of ~200 *E. affinis* copepodites (stages 4–5) was created by size-fractionating organisms using the artificial cohort method^27^. Briefly, a tall 300 µm sieve apparatus was lowered into a bucket filled with copepods from our cultures. Water inside the sieve apparatus was siphoned into a second bucket (<300 µm copepods). A tall 250 µm sieve apparatus was then lowered into this second bucket, and water inside the apparatus was siphoned out, leaving the desired copepodites (250 µm–300 µm) in the second bucket outside of the sieve apparatus. Copepodites were starved for four hours, and the experiment was initiated within 2 hours of field water sample collection by introducing copepodites to sieved field water in an acid-washed 10-L polypropelene bottle (~30 copepods L^−1^). For the duration of the experiment, the bottle was kept at 20 ± 2 °C in the dark to prevent photodegradation, with continuous aeration and gentle orbital shaking to ensure mixing.

Copepod and particulate samples were taken before introducing copepods to field water and four days post-introduction. At each sampling point, copepods were collected, preserved in 30% ethanol, and frozen at −80 °C (150 to 300 individuals for lignin analyses, 5 individuals for metagenomic analyses). Also at each sampling point, a churn splitter was used to withdraw 300–500 mL aliquots of water. Aliquots were filtered through 0.3 µm glass fiber filters (Advantec) for particulate chlorophyll *a* analyses and through 0.45 µm HAWP nitrocellulose filters (Millipore) for lignin and metagenomic analyses.

### Chemical biomarker analyses

Chlorophyll *a* was measured spectrophotometrically (Hewlett-Packard photo-diode array, model 8453) using EPA method 446 and Lorenzen’s spectrophotometric equations^28,29^. Copepod and particulate lignin analyses were performed using CuO oxidation as in Hedges & Ertel^30^ with modifications outlined in Spencer *et al*.^31^ and Hernes *et al*.^32^. Briefly, samples were oxidized in 8% NaOH in the presence of excess CuO at 155 °C followed by acidification and ethyl acetate extraction. The extracted fraction was dried under a gentle stream of ultrapure nitrogen and trimethylsilyl derivitized with bis(trimethylsilyl)trifluoroacetamide (BSTFA) after redissolution in pyridine. Lignin phenols were separated using an Agilent 6890 gas chromatograph fitted with a DB5-MS capillary column (30 m, 0.25 mm inner diameter, J&W Scientific) and attached to an Agilent 5975 mass selective detector. Quantification was achieved using selective ion monitoring, standardization to an internal standard (cinnamic acid), and a five point calibration scheme of Hernes *et al*.^33^. Samples were blank-corrected due to trace lignin in reagents.

### Metagenomic analysis

Genomic DNA (gDNA) was extracted from each copepod and filtered particulates (one quarter of each filter) using a plant DNA extraction kit (NucleoSpin Plant II, Macherey-Nagel) according to the manufacturer’s protocol. Copepod gDNA was pooled and concentrated by ethanol precipitation^34^.

A set of custom primers, ITS2-modified1 (5′-TTTCGCTGCGTTCTTCATCG-3′) and ITS5-modified1 (5′-GGAAGGAGAAGTCGTAACAAGG-3′), was developed based on White *et al*.^35^ and targets the internal transcribed spacer region 1 (ITS1) of vascular plants and phytoplankton. Our preliminary data indicate that the primer set amplified the ITS1 from a wide range of vascular plants and phytoplankton at the sampling site (Supplementary Fig. S1). The primers used for PCR contained dual 8 bp indices, Illumina TruSeq sequencing primer binding sites, and the P5 and P7 Illumina adapters. PCR was performed using 5PRIME HotMasterMix (2.5x, QuantaBio), forward and reverse primers (final concentration: 0.1 µM), and bovine serum albumin (final concentration: 0.4 mg mL^−1^). The PCR cycling condition for particulate samples was as follows: initial denaturation step of 94 °C for 3 minutes, 25 cycles of 94 °C for 45 seconds, 60 °C for 60 seconds, and 72 °C for 90 seconds, followed by a final extension step at 72 °C for 10 minutes. The PCR cycling condition for copepod samples was identical except for an additional 5 cycles (30 cycles total) necessary because of weak signals with 25 cycles. The PCR product was separated on 2% agarose gel and observed by a transilluminator after staining with Gel Red staining dye (Biotium) for 30 minutes. All PCR was performed in duplicate and pooled to obtain sufficient DNA concentrations for sequencing reactions.

Sequencing reactions were performed by the Vincent J. Coates Genomics Sequencing Laboratory (GSL) at the University of Berkeley (http://qb3.berkeley.edu/gsl/). See Supplementary Methods for indexing, pooling, clean-up libraries, and sequencing reaction conditions.

Bioinformatics data analysis was performed using USEARCH v. 11 (drive5.com/usearch). Sequences were merged to assemble paired-end reads, quality filtered, clustered to operational taxonomic units (OTUs) with a threshold of 97% using a default data analysis pipeline^36^. Annotation of OTUs was performed by running DNA sequence similarity searches using the BLASTN program (v. 2.8.0) with the non-redundant nucleotide database downloaded from the NCBI GenBank website (https://www.ncbi.nlm.nih.gov/genbank/) on July 26^th^, 2018 (55,184,812 nucleotide sequences were available in the database). The BLASTN search results were subjected to removal of low similarity hits using a bitscore of 150 as a threshold. All data processing was performed using a custom workstation built with 2x Xeon E5-2630 6 core CPU with 256GB ECC RAM, 4x HDD in RAID 10 configuration for data storage, 64-bit Linux system with Ubuntu v. 16.04 LTS.

### Survival feeding experiment with food regimens

To measure the impact of specific autotrophic food sources on copepod longevity, *E. affinis* survival was monitored under food regimens that ranged in quality (pure diets or mixtures of phytoplankton and plant litters) and quantity (none, low, or average feeding rates, based on organism carbon requirements) (Table 1). Food types included live algae (*Chlorella* sp.) and several vascular plant litters representative of the Sacramento River Valley and estuarine wetlands (mixed annual grasses, cattails, and tule). Prior to the experiment, we performed preliminary studies of a similar design to determine species and mixture ratios of interest (data not shown).

**Table 1:** Quality (food type) and quantity (feeding rate) of daily food regimens for survival feeding experiments.

Treatment	Feeding rate^a^	Amount of carbon administered by food type	
Pure algae	Average	2.38 µg C (*Chlorella, algae*)	
Pure grass^b^	Average	2.38 µg C (Mixed grasses)	
Pure cattail^c^	Average	2.38 µg C (*Typha*, cattail)	
Pure tule^b^	Average	2.38 µg C (*Schoenoplectus*, tule)	
No food	N/A	N/A	
Pure algae	Low	N/A	0.6 µg C (*Chlorella, algae*)
Mixed-grass	Average	1.78 µg C (Mixed grasses)	0.6 µg C (*Chlorella, algae*)
Mixed-cattail	Average	1.78 µg C (*Typha*, cattail)	0.6 µg C (*Chlorella, algae*)
Mixed-tule	Average	1.78 µg C (*Schoenoplectus*, tule)	0.6 µg C (*Chlorella, algae*)

^a^Daily feeding rate is carbon-normalized across regimens regardless of food type. Average feeding rate, based on Kayfetz & Kimmerer^39^ is 2.38 µg C, and low feeding rate is 0.6 µg C.

^b^Mixed annual grasses were collected from the Sierra Foothills Research and Extension Center in California.

^c^Macrophytes were collected from SFE wetlands.

*Chlorella* was cultured at 25 °C under constant cool white fluorescent lighting with aeration in 10 L of CB media adjusted to pH 9^37^. After more than one week of growth, algal cells were harvested by settling for 5 days at 4 °C and decanted off media to make a concentrated algae slurry at approximately 1 × 10^8^ cells mL^−1^, then adjusted to a concentration of 1 × 10^6^ cells mL^−1^ in 2 ppt reconstituted water. Plant tissues were collected at the Sierra Foothills Research and Extension Center in California (grass) and SFE wetlands (tule and cattails). After collection, litter samples were rinsed, oven-dried, finely ground, and sieved to <63 µm as in Heinle *et al*.^38^ before suspending in 2 ppt reconstituted water.

To initiate the experiment, copepods raised in 2 ppt reconstituted water were size-fractionated to 250–300 µm using the artificial cohort method as described above, and a single copepodite was distributed into each well of twelve-well plates (Corning, New York) in 5 mL 2 ppt reconstituted water (maximum well volume 6.9 mL). Food was administered daily, and each treatment was carbon-normalized at 2.38 or 0.6 µg carbon per copepod for average and low feeding rates, respectively, according to the elemental composition of each algae or litter (Table 2). This carbon requirement is based on ingestion rates by a similar calanoid copepod (*Pseudodioptomus forbesi*) and was verified in preliminary experiments as an optimal quantity for *E. affinis* in this size class^39^. Throughout the duration of the experiment, plates were kept under low light in a room with a temperature of 20 ± 2 °C, and water was replaced every four days. Copepods were observed daily and survival rate was recorded until all copepods were deceased. Copepods were considered living if any movement was detected during observation.

**Table 2:** Elemental composition (C and N) of algae and vascular plant litters.

	Weight % C	Weight % N
Algae (*Chlorella* sp.)	25	5
Mixed grasses^a^	39	0.8
Cattail (*Typha latifolia*)^b^	42	0.4
Tule (*Schoenoplectus acutus*)^b^	40	0.4

^a^Mixed annual grasses were collected from the Sierra Foothills Research and Extension Center in California.

^b^Macrophytes were collected from SFE wetlands.

Nonparametric survival analysis was performed using the ‘Survival’ package in R (v. 3.5.1) to calculate a Kaplan-Meier estimator for each treatment and plot its associated Kaplan-Meier curve^40^. All treatments were performed in at least triplicate, with one replicate defined as one twelve-well plate (i.e. twelve copepods constituted a curve, with at least three curves per treatment). Curves were tested for difference using the log-rank test, with p < 0.05 designated as significantly different.

## Results

### Consumption feeding experiment with estuary water

#### Chemical biomarkers

Chlorophyll *a* and lignin analyses demonstrated simultaneous consumption of phytoplankton and vascular plant material, respectively (Table 3). Ambient water column chlorophyll *a* concentration was 3.4 ± 0.8 µg L^−1^. Given a copepod density of 30 copepods L^−1^ and a C:Chl *a* ratio of 35 (previously published for this region)^4,41^, this chlorophyll *a* concentration allowed for a total of 4 µg chlorophyll *a* C per copepod. During the four-day experiment, chlorophyll *a* concentrations decreased to 2.0 ± 0.3 µg L^−1^. Carbon-normalized yields of lignin concurrently increased in the water column, reflecting the decrease in labile phytoplankton concentrations. Vascular plants in the water column were dominated by non-woody angiosperms, as indicated by lignin syringyl:vanillyl (S:V) and cinnamyl:vanillyl (C:V) phenol ratios (non-woody angiosperm S:V and C:V range from 0.6–1.7 and 0.4–0.8, respectively)^42^. Lignin in copepods themselves increased during the experiment from 0.038 ng copepod^−1^ (likely due to trace lignin in culture media) to 1.9 ng copepod^−1^, indicating uptake of tPOC.

**Table 3:** Chemical biomarkers (chlorophyll *a* and lignin) in water column particulates and ingested copepod gut before and after consumption feeding experiment.

	Particulates (water column)				Ingested (copepod gut)		
	Chlorophyll *a* (µg L^−1^)	Lignin			Lignin		
		S:V^a^	C:V^b^	Λ8 (mg 100 mgOC^−1^)^c^	S:V^a^	C:V^b^	Normalized (ng copepod^−1^)
Before feeding	3.4 ± 0.8	1.3	0.38	0.99	0.68	0.44	0.038
After feeding	2.0 ± 0.3	0.97	0.38	1.3	1.3	N.D.^d^	1.9

^a^Ratio of syringyl lignin phenols (syringaldehyde, acetosyringone, syringic acid) to vanillyl lignin phenols (vanillin, acetovanillone, vanillic acid).

^b^Ratio of cinnamyl lignin phenols (p‐coumaric acid, ferulic acid) to vanillyl lignin phenols (vanillin, acetovanillone, vanillic acid).

^c^Carbon-normalized yield of eight lignin phenols (vanillin, acetovanillone, vanillic acid, syringaldehyde, acetosyringone, syringic acid, p‐coumaric acid, ferulic acid).

^d^N.D. = non-detect; lower than the instrument/method detection limit.

#### DNA metagenomics

Metagenomic analysis identified 776 (one singleton removed) individual operational taxonomic units (OTUs), 13 of which were successfully identified as autotrophic vascular plants, and 83 of which were identified as non-vascular autotrophs. Non-target OTUs – consisting of fungi, bacteria, cyanobacteria, and copepods – were excluded from the analysis because their amplification and identification could not be validated given the primers used in the study.

**Figure 1:**
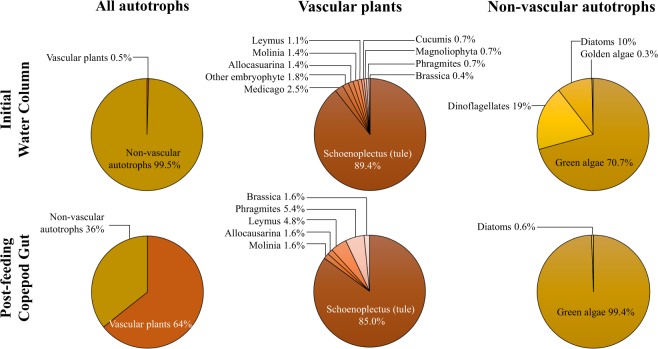
Percentage of internal transcribed spacer (ITS) sequences attributed to autotrophs in initial water column particulates (“initial water column”, top panels) and copepod gut after four days of feeding (“post-feeding copepod gut”, bottom panels). Left-most pie charts indicate relative percentages of total vascular and non-vascular autotrophs, while center and right-most pie charts show detailed distributions of each class.

While less than 1% of autotrophic ITS sequences identified in the initial water column were vascular plant-derived, vascular plants constituted more than half of autotrophic ITS sequences detected in *E. affinis* gut after four days of feeding (Fig. 1). Metagenomic analysis supported lignin data suggesting a dominance of angiosperms in the water column.

*Schoenoplectus sp*. (tule), a reed common to the study site, constituted the majority of vascular plant-derived ITS sequences in both the initial water column (89.4%) and copepod gut (85.0%) post-feeding, with other angiosperms contributing no more than 15%. Green algae comprised 70.7% non-vascular autotrophic ITS sequences in the water column with other notable contributions from diatoms (10%) and dinoflagellates (19%), but copepod gut post-feeding was almost completely dominated (99.4%) by green algae (Fig. 1).

### Survival feeding experiment with food regimens

While the previous experiment was aimed at food quantity (i.e. relative consumption of autotrophs by copepods), this experiment was designed to assess the impact of food quality, comparing the survival of copepods under conditions that included no food, pure phytoplankton, pure tPOC, and various mixtures of phytoplankton and tPOC.

**Figure 2:**
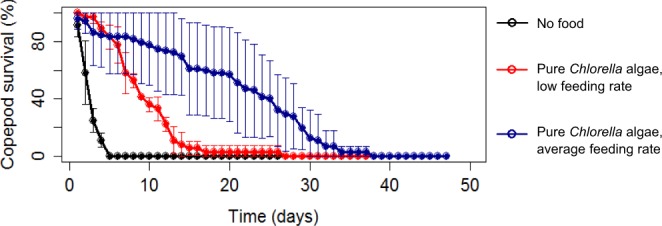
*E. affinis* survival curves for pure *Chlorella* algae food treatments at a low feeding rate (red, 0.6 µg C d^−1^) and average feeding rate (blue, 2.38 µg C d^−1^). A no food control (black, 0 µg C d^−1^) is plotted for reference.

On average, copepods could survive 4 days under conditions of no food, 18 days when fed pure phytoplankton at a low rate (0.6 µg C d^−1^), and 32 days when fed phytoplankton at an average rate (2.38 µg C d^−1^) (Fig. 2). Survivability log-rank tests indicated no difference in survival between a pure vascular plant diet and the ‘no food’ negative control (p < 0.05).

**Figure 3:**
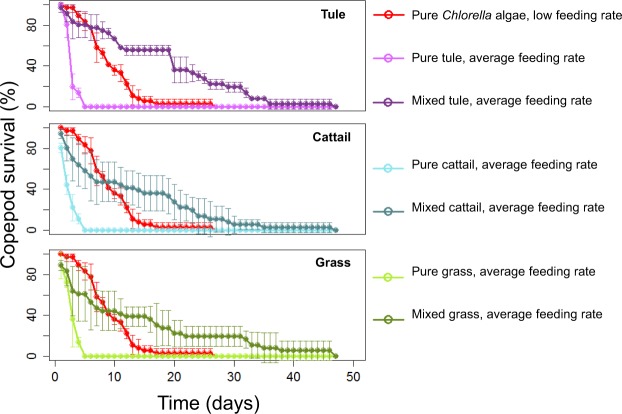
*E. affinis* survival curves for algal and vascular plant treatments. Vascular plants consisted of macrophytes (tule & cattail from the estuary) and grasses (mixed species from the catchment area). For each of tule (purple, top panel), cattail (blue, center panel), and grass (green, bottom panel), average feeding rate survival curves (2.38 µg C d^−1^) are plotted for pure plant material (lighter colors) and mixed algae and plant material (1:3 carbon ratio, darker colors). Pure *Chlorella* at a low feeding rate (red, 0.6 µg C d^−1^) is plotted for reference.

Survival in mixed treatments was generally less than that of the pure algae treatment at an average feeding rate but greater than that of the pure algae treatment at a low feeding rate (Fig. 3). For mixed tule, cattail, and grass treatments, survival was less than or equal to that of the pure algae at an average feeding rate in 78%, 89%, and 83% of cases, respectively (p < 0.05). Compared to the pure algae treatment at a low feeding rate, survival was strongly (100% of cases) enhanced in the mixed tule treatment and weakly (33% of cases) enhanced in the mixed cattail and mixed grass treatments (p < 0.05) (Fig. 3).

## Discussion

Macrophyte- and terrestrial-derived detritus is not only a major contributor to the OM pool in estuaries, but also plays a vital role in the lower aquatic food web mediated by active uptake by zooplankton, even when algae is accessible. In the estuarine water consumption feeding experiments, chlorophyll *a* concentrations decreased only 41%, suggesting that phytoplankton resources were available throughout the experiment. Nevertheless, we observed a concurrent increase in copepod gut lignin content, indicating that copepods were consuming plant detritus.

Copepod consumption of plant detritus was disproportionately higher than the amount of tPOC present in the water column, an indication that copepods were actively – not passively or accidentally – feeding on detritus. Water column lignin yields were relatively low (near 1 mg 100 mgOC^−1^ compared to 2.5 ± 2 mg 100 mgOC^−1^ for coastal US rivers)^43^ and terrestrial detritus accounted for only 0.5% of all autotrophic ITS sequences in the water column, and yet it was an appreciable component of copepod gut content (64% of autotrophic ITS sequences).

The disproportion between the water column autotrophic composition and that of the copepod gut supports well-established discriminatory feeding behavior ascribed to *E. affinis*. However, while several studies indicate preference for live phytoplankton over detritus (which includes non-living phytoplankton)^44–46^ this study suggests they are selecting for vascular plant detrital material rather than against it. Notably, DNA metagenomics cannot distinguish between living and non-living phytoplankton, so a considerable proportion of non-vascular autotrophic sequences may have been non-living, and potentially less desirable than if all phytoplankton were living. Yet chlorophyll *a* concentrations remained high throughout the experiment, suggesting that living phytoplankton – dominated by green algae (71% of non-vascular autotrophic ITS sequences) – were in supply for the duration of the experiment.

The chemical composition of vascular plant particulates demonstrates its inferiority as a direct food resource relative to phytoplankton (particularly green algae). Vascular plants possess only 2–5% C as protein and <3% C as lipids, compared to 25–50% protein C and 5–20% lipid C for phytoplankton^9^, and they lack essential fatty acids^10^. Instead, vascular plant material contains stable lignocellulose complexes that are energetically unfavorable to degrade^47–49^ and carbohydrates that are easily degradable but have low energy density^50^. The proof of this low quality was exhibited in the survival experiments in which treatments of vascular plant carbon alone equated to ‘no food’ treatments, likely due to the lack of essential fatty acids.

Accessibility of terrestrially-derived nutrients to copepods appeared to be coupled with the presence or absence of phytoplankton, similar to observations from *D. magna* growth rate studies^13^. While a pure algae diet sustained copepods for up to 30 days, a pure vascular plant diet was nutritionally insufficient to sustain copepods for more than a few days. Of course, the composition of estuarine waters is complex and at any given time zooplankton are expected to encounter any number of food sources. Mixture treatments of algae and plant material enhanced *E. affinis* survival over the pure algae treatment at a low feeding rate, demonstrating a biological motive for deliberate consumption of plant material in the presence of phytoplankton.

The presence of plant material increased copepod longevity (i.e. lengthened the tail of survival curves) for all mixture treatments relative to pure algae, but the extent to which tPOC enhanced overall survival was dependent on plant species. The strongest enhancement was found in treatments with *Schoenoplectus* sp. (tule), a common sedge that dominates our study site in the SFE and is found in marshes, swamps, and shallow water areas throughout North America and Eurasia. For a vascular plant, tule consists of average to above-average nutritional quality with a crude protein content of approximately 10%^51^, and both a lower lignin content and slightly higher nutrient content (N, P) than similar wetland plants (*Phragmites spp*. – common reed and *Typha spp*. – cattail)^52^. Yet it is still nutritionally inferior to green algae. *Nannochloropsis* algae, for example, has a crude protein content of nearly 30% and lipid content of 18%, with almost half of its fatty acids attributed to essential polyunsaturated fatty acids such as eicosapentanoic acid (EPA) and docosahexanoic acid (DHA)^53,54^. Crude protein content in *Chlorella* can reach 55%, with PUFAs comprising approximately 70% of fatty acid composition^54,55^.

One explanation for the apparent discrepancy between vascular plant consumption and nutritional content is that it is not the plant material itself that is being utilized but that vascular plant particulates act as a substrate upon which bacteria can colonize^38,56,57^. While there is some rationale for this given the favorable nutritional profile of bacteria^58,59^, measurable bacterial contributions to copepod survival are unlikely in our study for several reasons. First, based on bacterial counts on plant materials prepared similarly to those in this study^60^ and based on the typical carbon content of bacterial cells^61^, the total bacterial carbon likely constitutes <0.00001% of the carbon administered in any of the food treatments. Second, if colonizing bacteria were the sole reason for enhanced survival, the pure vascular plant diet should have stimulated growth. Pure vascular plant treatments proved inadequate for survival and were comparable only to ‘no food’ controls. Third, the differential results between the three plant materials is most easily explained by differences in the plant compositions themselves. A primarily bacterial nutrient source would require that each plant material possess a fundamentally different bacterial community composition with an appreciably different biochemical profile.

If nutritional benefits are conferred directly from the plant material, they may be attributed to co-metabolism, where the presence of labile particulates aids in the degradation of more nutrient-poor particulates^11^. Fatty acid analyses from *D. magna* feeding experiments suggest that vascular plant-derived metabolites may be used for regular energy needs in order for algal-derived nutrients (high in EPA and DHA) to be reserved for growth^13^. In this study, enhanced survival may be attributed to a similar compartmentalization system, where plant material provided life-sustaining metabolites to increase copepod longevity over copepods fed pure algae at the lower feeding rate.

The SFE allows for a ‘perfect storm’ scenario for co-metabolism, given low *in situ* primary production, substantial tPOC inputs, and a dominance of highly selective copepods in the water column. Co-metabolism may be particularly relevant during winter storms and spring snowmelt, where high riverine flows flush terrestrially-derived particulates into the water column and perturb *in situ* macrophytes, providing a fresh source of particulates and limiting photic depth necessary for phytoplankton growth.

In addition to seasonal impacts, landscape diversity influences whether tPOC stimulates the lower aquatic food web. The SFE has sustained major landscape changes in the past century; an estuary that was once a nutrient-deplete, terrestrial-replete marsh system has been transformed into a nutrient-replete, terrestrial-deplete system of leveed and drained islands^62^. If ties were once strong between tPOC and the lower food web in the marsh-dominated SFE, they have surely weakened after such landscape modifications. Yet even in present-day SFE conditions we find evidence that tPOC is impactful in supporting the lower food web.

Tidal marsh restoration is becoming commonplace in the SFE and other estuaries as policies have evolved to recognize the range of benefits conferred by wetland habitat^63,64^. While specific goals of these restoration projects vary, in most cases vegetation cover expands through the creation of tidal and non-tidal wetlands, invariably increasing the contribution of tPOC to these estuaries.

Since the significance of tPOC to zooplankton diet is dependent on plant type, regionalized assessments of linkages between plant diversity and the pelagic food web are critical in directing restoration efforts that provide local food web benefits. In this study, for example, tule dominated in both distribution in the water column and survivability for *E. affinis*, proving to be a significant source of detrital-derived energy. In the Chesapeake Bay however, wild rice (*Zizania aquatica*) is a more pertinent plant species than tule that may contribute to copepod diet^38^. For the Chesapeake Bay and other estuaries, genetics is a new tool to add to the toolkit, with tremendous potential to contribute a plant-specific linkage of macrophytic or terrestrial-derived wetland POC to localized food webs.

In summary, we demonstrate unequivocally that copepods consume plant detritus, selecting for tPOC even when phytoplankton are available. Uptake of tPOC is deliberate, enhancing copepod survival and perhaps providing population resilience in times and places where algal food resources are insufficient. Marshes are one such place that can be limited in autochthonous production but deliver substantial amounts of terrestrial or macrophytic detritus to the estuarine water column, providing benefits to pelagic food webs that have previously been overlooked. Notably, though, the extent to which terrestrial material can enhance copepod survival is dependent on plant type and may therefore be specific to local ecosystems. Metagenomic analysis holds promise as a highly sensitive new tool that can be optimized in any localized landscape to explore interactions between regional tPOC inputs and pelagic food webs.

## Supplementary information

Supplementary Information

## Data Availability

The metagenomic dataset generated during the current study is available in the NCBI GenBank database (BioProject ID: PRJNA504478; BioSample accession numbers: SAMN10393053, SAMN10393054). All other data are available from the corresponding author on reasonable request.
