# Supplementary material for: Plant detritus is selectively consumed by estuarine copepods and can augment their survival
Source: Sci Rep. 2019 Jun 24;9:9076. doi: 10.1038/s41598-019-45503-6 (PMC6591215; doi:10.1038/s41598-019-45503-6)
Supplement: Supplementary file 1 — Supplementary Information [file 41598_2019_45503_MOESM1_ESM.pdf]

Plant detritus is selectively consumed by estuarine copepods and can augment their survival

Jennifer Harfmann[1*], Tomofumi Kurobe[2], Brian Bergamaschi[3], Swee Teh[2], and Peter Hernes[1]

[1]Department of Land, Air, and Water Resources, University of California, Davis, CA USA

[2]Department of Anatomy, Physiology, and Cell Biology, School of Veterinary Medicine, University of California, Davis, CA USA

[3]United States Geological Survey, Sacramento, CA USA

*Corresponding author

Email: jharfman@ucdavis.edu

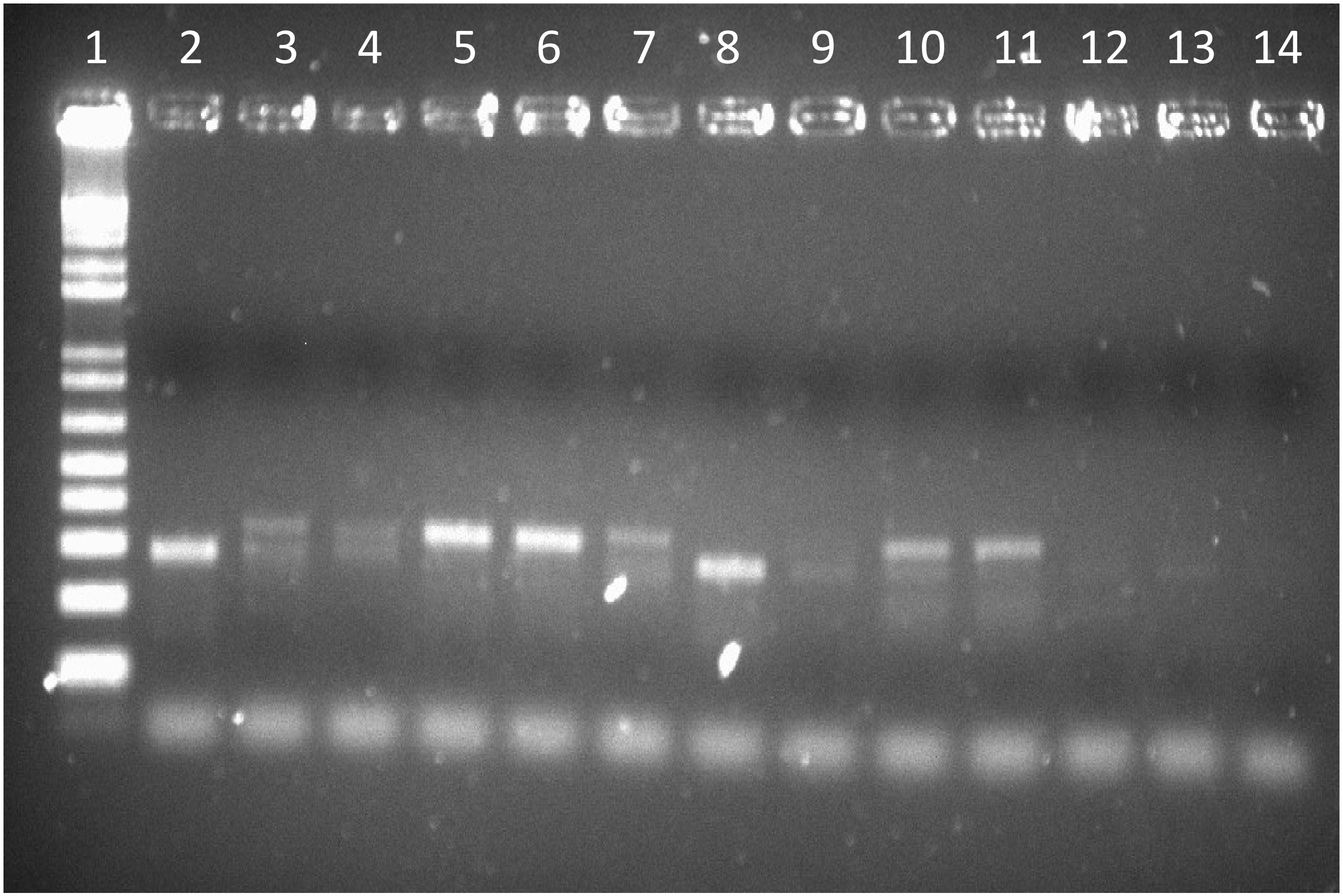

Supplementary Figure S1. PCR gel testing primers ITS2-modified1 and ITS5-modified1 with vascular plants (tule, lanes 2 & 8; cattail, lanes 3 & 7; common reed, lanes 4 & 9; rye grass, lanes 5 & 6; and pampas grass, lanes 10 & 11) and mixed algae (lanes 12 & 13) from the sampling site. Lanes 1 and 14 correspond to 1 Kb Plus DNA Ladder (Thermo Fisher Scientific) and negative control, respectively.

Supplementary Methods

Amplicon metagenomic sequencing reaction conditions (to accompany Methods section 2:ii: Metagenomic analyses)

Indexing PCR was performed in 25 µL reactions using 10 µL 5PRIME HotMasterMix (2.5x, QuantaBio), 5 µL molecular grade H_2O, 5 µL template DNA, and 2 µL each of a premixed (containing both forward and reverse) TruSeq adapter primer set with 8 bp unique dual indexes (10 µM). Cycling conditions for the indexing reactions were 94 °C for 3 minutes, 8 cycles of 94 °C for 45 seconds, 52 °C for 60 seconds, and 72 °C for 90 seconds, with a final extension of 72 °C for 10 minutes. Successful indexing PCR, as judged by an average fragment size of 405 bp and reasonable concentration per reaction, was confirmed using an Agilent AATI Fragment Analyzer. Individual samples were pooled equimolarly based on DNA concentrations from the Fragment Analyzer for the target amplicon size and the final pool was cleaned using Agencourt AMPure beads (Beckman Coulter) with a 0.8x bead to pool volume ratio. The final, cleaned pool was quantified in duplicate using the Library Quantification qPCR Kit (KAPA Biosystems) and sequenced at 11pM with Illumina MiSeq 300PE v3 chemistry (Illumina). No extra base-diversity generating PhiX spike-in was required as modifications were incorporated into the initial PCR primer design. Resultant BCL files were then converted to FASTQ format using Illumina's bcl2fastq v2.19 software (https://support.illumina.com/downloads/bcl2fastq-conversion-software-v2-19.html). Raw FASTQ files were then demultiplexed and initial PCR target primer binding sites were removed using the dbcAmplicons software suite developed by the UC Davis Bioinformatics Core (unpublished protocol, found here: https://ucdavis-bioinformatics-training.github.io/2017-September-Microbial-Community-Analysis-Workshop/thursday/dbcAmplicons_Bioinformatics.pdf).
